# Supplementary material for: Resolving the full spectrum of human genome variation using Linked-Reads
Source: Genome Res. 2019 Apr;29(4):635–45. doi: 10.1101/gr.234443.118 (PMC6442396; doi:10.1101/gr.234443.118)
Supplement: Supplemental Material [file supp_gr.234443.118_Supplemental_longranger-2.2.zip › longranger-2.2.tar.bz2!/lr2.2-public/lib/stan/cmdstan/stan/doc/api/html/stan__csv__reader_8hpp_source.html]

Stan: src/stan/io/stan\_csv\_reader.hpp Source File


|  |  |
| --- | --- |
|  | Stan  2.5.0  probability, sampling & optimization |


- Main Page
- Related Pages
- Namespaces
- Classes
- Files

- File List
- File Members

All Classes Namespaces Files Functions Variables Typedefs Enumerations Enumerator Friends Macros Pages

- src
- stan
- io

stan\_csv\_reader.hpp

Go to the documentation of this file.

1 #ifndef STAN\_\_IO\_\_STAN\_CSV\_READER\_HPP

2 #define STAN\_\_IO\_\_STAN\_CSV\_READER\_HPP

3

4 #include <istream>

5 #include <iostream>

6 #include <sstream>

7 #include <string>

8 #include <boost/algorithm/string.hpp>

9 #include <boost/lexical\_cast.hpp>

10 #include <stan/math/matrix.hpp>

11

12 namespace stan {

13  namespace io {

14

15  // FIXME: should consolidate with the options from the command line in stan::gm

16  struct stan\_csv\_metadata {

17  int stan\_version\_major;

18  int stan\_version\_minor;

19  int stan\_version\_patch;

20

21  std::string model;

22  std::string data;

23  std::string init;

24  size\_t chain\_id;

25  size\_t seed;

26  bool random\_seed;

27  size\_t num\_samples;

28  size\_t num\_warmup;

29  bool save\_warmup;

30  size\_t thin;

31  bool append\_samples;

32  std::string algorithm;

33  std::string engine;

34

35  stan\_csv\_metadata()

36  : stan\_version\_major(0), stan\_version\_minor(0), stan\_version\_patch(0),

37  model(""), data(""), init(""),

38  chain\_id(1), seed(0), random\_seed(false),

39  num\_samples(0), num\_warmup(0), save\_warmup(false), thin(0),

40  append\_samples(false),

41  algorithm(""), engine("") {}

42  };

43

44  struct stan\_csv\_adaptation {

45  double step\_size;

46  Eigen::MatrixXd metric;

47

48  stan\_csv\_adaptation()

49  : step\_size(0), metric(0,0) {}

50  };

51

52  struct stan\_csv\_timing {

53  double warmup;

54  double sampling;

55

56  stan\_csv\_timing()

57  : warmup(0), sampling(0) {}

58  };

59

60  struct stan\_csv {

61  stan\_csv\_metadata metadata;

62  Eigen::Matrix<std::string, Eigen::Dynamic, 1> header;

63  stan\_csv\_adaptation adaptation;

64  Eigen::MatrixXd samples;

65  stan\_csv\_timing timing;

66  };

67

71  class stan\_csv\_reader {

72

73  public:

74

75  stan\_csv\_reader() {}

76  ~stan\_csv\_reader() {}

77

78  static bool read\_metadata(std::istream& in, stan\_csv\_metadata& metadata) {

79

80  std::stringstream ss;

81  std::string line;

82

83  if (in.peek() != '#')

84  return false;

85  while (in.peek() == '#') {

86  std::getline(in, line);

87  ss << line << '\n';

88  }

89  ss.seekg(std::ios\_base::beg);

90

91  char comment;

92  std::string lhs;

93

94  std::string name;

95  std::string value;

96

97  while (ss.good()) {

98

99  ss >> comment;

100  std::getline(ss, lhs);

101

102  size\_t equal = lhs.find("=");

103  if (equal != std::string::npos) {

104  name = lhs.substr(0, equal);

105  boost::trim(name);

106  value = lhs.substr(equal + 2, lhs.size());

107  boost::replace\_first(value, " (Default)", "");

108  } else {

109  continue;

110  }

111

112  if (name.compare("stan\_version\_major") == 0) {

113  metadata.stan\_version\_major = boost::lexical\_cast<int>(value);

114  } else if (name.compare("stan\_version\_minor") == 0) {

115  metadata.stan\_version\_minor = boost::lexical\_cast<int>(value);

116  } else if (name.compare("stan\_version\_patch") == 0) {

117  metadata.stan\_version\_patch = boost::lexical\_cast<int>(value);

118  } else if (name.compare("model") == 0) {

119  metadata.model = value;

120  } else if (name.compare("num\_samples") == 0) {

121  metadata.num\_samples = boost::lexical\_cast<int>(value);

122  } else if (name.compare("num\_warmup") == 0) {

123  metadata.num\_warmup = boost::lexical\_cast<int>(value);

124  } else if (name.compare("save\_warmup") == 0) {

125  metadata.save\_warmup = boost::lexical\_cast<bool>(value);

126  } else if (name.compare("thin") == 0) {

127  metadata.thin = boost::lexical\_cast<int>(value);

128  } else if (name.compare("chain\_id") == 0) {

129  metadata.chain\_id = boost::lexical\_cast<int>(value);

130  } else if (name.compare("data") == 0) {

131  metadata.data = value;

132  } else if (name.compare("init") == 0) {

133  metadata.init = value;

134  boost::trim(metadata.init);

135  } else if (name.compare("seed") == 0) {

136  metadata.seed = boost::lexical\_cast<unsigned int>(value);

137  metadata.random\_seed = false;

138  } else if (name.compare("append\_samples") == 0) {

139  metadata.append\_samples = boost::lexical\_cast<bool>(value);

140  } else if (name.compare("algorithm") == 0) {

141  metadata.algorithm = value;

142  } else if (name.compare("engine") == 0) {

143  metadata.engine = value;

144  }

145

146  }

147

148  if (ss.good() == true)

149  return false;

150

151  return true;

152

153  } // read\_metadata

154

155  static bool read\_header(std::istream& in, Eigen::Matrix<std::string, Eigen::Dynamic, 1>& header) {

156

157  std::string line;

158

159  if (in.peek() != 'l')

160  return false;

161

162  std::getline(in, line);

163  std::stringstream ss(line);

164

165  header.resize(std::count(line.begin(), line.end(), ',') + 1);

166  int idx = 0;

167  while (ss.good()) {

168  std::string token;

169  std::getline(ss, token, ',');

170  boost::trim(token);

171

172  int pos = token.find('.');

173  if (pos > 0) {

174  token.replace(pos, 1, "[");

175  std::replace(token.begin(), token.end(), '.', ',');

176  token += "]";

177  }

178  header(idx++) = token;

179  }

180  return true;

181  }

182

183  static bool read\_adaptation(std::istream& in, stan\_csv\_adaptation& adaptation) {

184

185  std::stringstream ss;

186  std::string line;

187  int lines = 0;

188

189  if (in.peek() != '#' || in.good() == false)

190  return false;

191

192  while (in.peek() == '#') {

193  std::getline(in, line);

194  ss << line << std::endl;

195  lines++;

196  }

197  ss.seekg(std::ios\_base::beg);

198

199  char comment; // Buffer for comment indicator, #

200

201  // Skip first two lines

202  std::getline(ss, line);

203

204  // Stepsize

205  std::getline(ss, line, '=');

206  boost::trim(line);

207  ss >> adaptation.step\_size;

208

209  // Metric parameters

210  std::getline(ss, line);

211  std::getline(ss, line);

212  std::getline(ss, line);

213

214  int rows = lines - 3;

215  int cols = std::count(line.begin(), line.end(), ',') + 1;

216  adaptation.metric.resize(rows, cols);

217

218  for (int row = 0; row < rows; row++) {

219

220  std::stringstream line\_ss;

221  line\_ss.str(line);

222  line\_ss >> comment;

223

224  for (int col = 0; col < cols; col++) {

225  std::string token;

226  std::getline(line\_ss, token, ',');

227  boost::trim(token);

228  adaptation.metric(row, col) = boost::lexical\_cast<double>(token);

229  }

230

231  std::getline(ss, line); // Read in next line

232

233  }

234

235  if (ss.good())

236  return false;

237  else

238  return true;

239

240  }

241

242  static bool read\_samples(std::istream& in, Eigen::MatrixXd& samples, stan\_csv\_timing& timing) {

243

244  std::stringstream ss;

245  std::string line;

246

247  int rows = 0;

248  int cols = -1;

249

250  if (in.peek() == '#' || in.good() == false)

251  return false;

252

253  while (in.good()) {

254

255  bool comment\_line = (in.peek() == '#');

256  bool empty\_line = (in.peek() == '\n');

257

258  std::getline(in, line);

259

260  if (empty\_line) continue;

261  if (!line.length()) break;

262

263  if (comment\_line) {

264

265  if (line.find("(Warm-up)") != std::string::npos) {

266  int left = 17;

267  int right = line.find(" seconds");

268  timing.warmup += boost::lexical\_cast<double>(line.substr(left, right - left));

269  } else if (line.find("(Sampling)") != std::string::npos) {

270  int left = 17;

271  int right = line.find(" seconds");

272  timing.sampling += boost::lexical\_cast<double>(line.substr(left, right - left));

273  }

274

275  }

276  else {

277

278  ss << line << '\n';

279

280  int current\_cols = std::count(line.begin(), line.end(), ',') + 1;

281  if (cols == -1) {

282  cols = current\_cols;

283  } else if (cols != current\_cols) {

284  std::cout << "Error: expected " << cols << " columns, but found "

285  << current\_cols << " instead for row " << rows + 1 << std::endl;

286  return false;

287  }

288  rows++;

289

290  }

291

292  in.peek();

293

294  }

295

296  ss.seekg(std::ios\_base::beg);

297

298  if (rows > 0) {

299  samples.resize(rows, cols);

300  for (int row = 0; row < rows; row++) {

301  std::getline(ss, line);

302  std::stringstream ls(line);

303  for (int col = 0; col < cols; col++) {

304  std::getline(ls, line, ',');

305  boost::trim(line);

306  //std::cout << "line: !" << line << "@" << std::endl;

307  samples(row, col) = boost::lexical\_cast<double>(line);

308  //std::cout << "after" << std::endl << std::endl;

309  }

310  }

311  }

312  return true;

313  }

314

319  static stan\_csv parse(std::istream& in) {

320

321  stan\_csv data;

322

323  if (!read\_metadata(in, data.metadata)) {

324  std::cout << "Warning: non-fatal error reading metadata" << std::endl;

325  }

326

327  if (!read\_header(in, data.header)) {

328  std::cout << "Error: error reading header" << std::endl;

329  throw std::invalid\_argument("Error with header of input file in parse");

330  }

331

332  if (!read\_adaptation(in, data.adaptation)) {

333  std::cout << "Warning: non-fatal error reading adapation data" << std::endl;

334  }

335

336  data.timing.warmup = 0;

337  data.timing.sampling = 0;

338

339  if (!read\_samples(in, data.samples, data.timing)) {

340  std::cout << "Warning: non-fatal error reading samples" << std::endl;

341  }

342

343  return data;

344

345  }

346

347  };

348

349  } // io

350

351 } // stan

352

353 #endif

stan::io::stan\_csv\_metadata::num\_samples

size\_t num\_samples

**Definition:** stan\_csv\_reader.hpp:27

stan::io::stan\_csv\_metadata::chain\_id

size\_t chain\_id

**Definition:** stan\_csv\_reader.hpp:24

stan::io::stan\_csv::samples

Eigen::MatrixXd samples

**Definition:** stan\_csv\_reader.hpp:64

stan::io::stan\_csv\_metadata

**Definition:** stan\_csv\_reader.hpp:16

stan::io::stan\_csv\_reader::read\_samples

static bool read\_samples(std::istream &in, Eigen::MatrixXd &samples, stan\_csv\_timing &timing)

**Definition:** stan\_csv\_reader.hpp:242

stan::io::stan\_csv\_metadata::data

std::string data

**Definition:** stan\_csv\_reader.hpp:22

stan::io::stan\_csv\_adaptation::metric

Eigen::MatrixXd metric

**Definition:** stan\_csv\_reader.hpp:46

stan::math::row

Eigen::Matrix< T, 1, Eigen::Dynamic > row(const Eigen::Matrix< T, Eigen::Dynamic, Eigen::Dynamic > &m, size\_t i)

Return the specified row of the specified matrix, using start-at-1 indexing.

**Definition:** row.hpp:26

stan::io::stan\_csv\_metadata::algorithm

std::string algorithm

**Definition:** stan\_csv\_reader.hpp:32

stan::io::stan\_csv\_metadata::init

std::string init

**Definition:** stan\_csv\_reader.hpp:23

stan::io::stan\_csv\_reader::read\_adaptation

static bool read\_adaptation(std::istream &in, stan\_csv\_adaptation &adaptation)

**Definition:** stan\_csv\_reader.hpp:183

stan::io::stan\_csv\_adaptation::stan\_csv\_adaptation

stan\_csv\_adaptation()

**Definition:** stan\_csv\_reader.hpp:48

stan::io::stan\_csv\_metadata::stan\_version\_patch

int stan\_version\_patch

**Definition:** stan\_csv\_reader.hpp:19

stan::io::stan\_csv\_reader::stan\_csv\_reader

stan\_csv\_reader()

**Definition:** stan\_csv\_reader.hpp:75

stan::io::stan\_csv\_metadata::random\_seed

bool random\_seed

**Definition:** stan\_csv\_reader.hpp:26

stan::math::col

Eigen::Matrix< T, Eigen::Dynamic, 1 > col(const Eigen::Matrix< T, Eigen::Dynamic, Eigen::Dynamic > &m, size\_t j)

Return the specified column of the specified matrix using start-at-1 indexing.

**Definition:** col.hpp:24

stan::io::stan\_csv\_metadata::engine

std::string engine

**Definition:** stan\_csv\_reader.hpp:33

stan::io::stan\_csv\_reader::read\_header

static bool read\_header(std::istream &in, Eigen::Matrix< std::string, Eigen::Dynamic, 1 > &header)

**Definition:** stan\_csv\_reader.hpp:155

stan::io::stan\_csv\_timing::warmup

double warmup

**Definition:** stan\_csv\_reader.hpp:53

stan::io::stan\_csv\_timing

**Definition:** stan\_csv\_reader.hpp:52

stan::io::stan\_csv::adaptation

stan\_csv\_adaptation adaptation

**Definition:** stan\_csv\_reader.hpp:63

stan::io::stan\_csv\_timing::stan\_csv\_timing

stan\_csv\_timing()

**Definition:** stan\_csv\_reader.hpp:56

stan::io::stan\_csv\_metadata::stan\_version\_minor

int stan\_version\_minor

**Definition:** stan\_csv\_reader.hpp:18

stan::io::stan\_csv\_metadata::thin

size\_t thin

**Definition:** stan\_csv\_reader.hpp:30

stan::math::rows

size\_t rows(const Eigen::Matrix< T, R, C > &m)

**Definition:** rows.hpp:12

stan::io::stan\_csv\_adaptation::step\_size

double step\_size

**Definition:** stan\_csv\_reader.hpp:45

stan::io::stan\_csv

**Definition:** stan\_csv\_reader.hpp:60

stan::io::stan\_csv\_metadata::stan\_csv\_metadata

stan\_csv\_metadata()

**Definition:** stan\_csv\_reader.hpp:35

stan::math::cols

size\_t cols(const Eigen::Matrix< T, R, C > &m)

**Definition:** cols.hpp:12

stan::io::stan\_csv\_reader::read\_metadata

static bool read\_metadata(std::istream &in, stan\_csv\_metadata &metadata)

**Definition:** stan\_csv\_reader.hpp:78

stan::io::stan\_csv\_metadata::num\_warmup

size\_t num\_warmup

**Definition:** stan\_csv\_reader.hpp:28

stan::io::stan\_csv\_metadata::model

std::string model

**Definition:** stan\_csv\_reader.hpp:21

stan::io::stan\_csv\_metadata::append\_samples

bool append\_samples

**Definition:** stan\_csv\_reader.hpp:31

stan::io::stan\_csv\_metadata::save\_warmup

bool save\_warmup

**Definition:** stan\_csv\_reader.hpp:29

stan::io::stan\_csv\_reader

Reads from a Stan output csv file.

**Definition:** stan\_csv\_reader.hpp:71

stan::io::stan\_csv\_reader::parse

static stan\_csv parse(std::istream &in)

Parses the file.

**Definition:** stan\_csv\_reader.hpp:319

stan::io::stan\_csv\_metadata::seed

size\_t seed

**Definition:** stan\_csv\_reader.hpp:25

stan::io::stan\_csv::metadata

stan\_csv\_metadata metadata

**Definition:** stan\_csv\_reader.hpp:61

stan::io::stan\_csv::header

Eigen::Matrix< std::string, Eigen::Dynamic, 1 > header

**Definition:** stan\_csv\_reader.hpp:62

stan::io::stan\_csv\_metadata::stan\_version\_major

int stan\_version\_major

**Definition:** stan\_csv\_reader.hpp:17

stan::io::stan\_csv\_adaptation

**Definition:** stan\_csv\_reader.hpp:44

stan::io::stan\_csv::timing

stan\_csv\_timing timing

**Definition:** stan\_csv\_reader.hpp:65

stan::io::stan\_csv\_reader::~stan\_csv\_reader

~stan\_csv\_reader()

**Definition:** stan\_csv\_reader.hpp:76

stan::io::stan\_csv\_timing::sampling

double sampling

**Definition:** stan\_csv\_reader.hpp:54

matrix.hpp


---

[ Stan Home Page ]

*© 2011–2014,
Stan Development Team.*
